# Over-Expression of p190RhoGEF Regulates the Formation of Atherosclerotic Plaques in the Aorta of ApoE^−/−^ Mice via Macrophage Polarization

**DOI:** 10.3390/ijms241612785

**Published:** 2023-08-14

**Authors:** So-Yeon Choi, Eun-Bi Lee, Jee-Hae Kim, Jong Ran Lee

**Affiliations:** 1 Department of Bioinspired Science, The Graduate School, Ewha Womans University, Seoul 03760, Republic of Korea; 2 Department of Life Science, College of Natural Sciences, Ewha Womans University, Seoul 03760, Republic of Korea

**Keywords:** macrophage, p190RhoGEF, inflammatory cytokines, atherosclerotic plaques, ApoE^−/−^ mice

## Abstract

The RhoA-specific guanine nucleotide exchange factor p190RhoGEF has been implicated in the control of cell morphology, focal adhesion formation, and cell motility. Previously, we reported that p190RhoGEF is also active in various immune cells. In this study, we examined whether over-expression of p190RhoGEF could affect atherosclerotic plaque formation in mouse aortae. For that purpose, transgenic (TG) mice over-expressing p190RhoGEF were cross-bred with atherosclerosis-prone apolipoprotein E (ApoE)^−/−^ mice to obtain p190RhoGEF-TG mice with ApoE^−/−^ backgrounds (TG/ApoE^−/−^). Aortic plaque formation was significantly increased in TG/ApoE mice^−/−^ at 30 to 40 weeks of age compared to that in ApoE^−/−^ mice. Serum concentrations of inflammatory cytokines (IL-6 and TNF-α) were greater in TG/ApoE^−/−^ mice than in ApoE^−/−^ mice at ~40 weeks of age. Furthermore, TG/ApoE^−/−^ mice had a greater proportion of peritoneal macrophages within the M1 subset at 30 to 40 weeks of age, together with higher production of inflammatory cytokines and stronger responses to bacterial lipopolysaccharide than ApoE^−/−^ mice. Collectively, these results highlight a crucial role of enhanced p190RhoGEF expression in atherosclerosis progression, including the activation of pro-inflammatory M1 macrophages.

## 1. Introduction

The guanine nucleotide exchange factor p190RhoGEF (also known as Rgnef) has been implicated in the control of neuronal morphology, degeneration, axonal branching, and synapse formation [1,2,3,4]. Elevated expression of p190RhoGEF has been shown to promote RhoA activity, focal adhesion formation, cell motility, and tumor progression [5,6]. We reported the role of this protein in several immune cells using transgenic (TG) mice, in which a transgene is specifically expressed in antigen-presenting cells bearing the class II MHC [7]. Our studies in TG mice showed that over-expression of p190RhoGEF enhances B cell activation, germinal center formation, and plasma cell differentiation during T cell-dependent humoral immune responses [8,9,10]. Studies in conventional CD11c^+^ dendritic cells (DCs) and macrophages of TG mice also showed that over-expression of p190RhoGEF negatively affects cellular responses to bacterial lipopolysaccharide (LPS). Impaired activation and localization of conventional DCs to the T cell zone in the spleen, as well as reduced interleukin (IL)-6 secretion, was seen in TG mice after LPS injection [11]. TG macrophages showed less polarization to the classical M1 subset population and consequently reduced inflammatory responses during LPS-elicited inflammation [12].

The development of atherosclerosis progresses due to the chronic arterial inflammation driven by immune cells and their mediators. Dyslipidemia, metabolic stress, and microbial infections activate inflammatory and immune mechanisms, which can promote the development and/or destabilization of atherosclerotic plaques [13,14,15,16]. Virtually major immune cell lineage has been identified in plaques, and the array of cytokines is implicated in atherosclerosis [15,16,17]. The roles of various components in immunity have been examined using atherosclerosis-prone low-density lipoprotein receptor (LDLR)^−/−^ or apolipoprotein E (ApoE)^−/−^ mice [18,19,20,21]. ApoE deficiency results in spontaneous atherosclerosis in standard rodent-chow-fed animals; these lesions occur most frequently in the proximal aorta and are characterized by vascular inflammation associated with infiltration of macrophages and other immune cells [18,19].

In particular, macrophages play various important roles in atherosclerosis. Macrophages are recruited to inflamed areas and acquire distinct functional phenotypes as either pro-inflammatory (M1) or anti-inflammatory (M2) macrophages [22,23,24]. Two distinct phenotypes of M1 and M2 macrophages represent different activation states with diverse functions and responses to stimuli: M1 macrophages are activated in response to pro-inflammatory stimuli, such as LPS and interferon (IFN)-γ. They secrete pro-inflammatory cytokines, including tumor necrosis factor (TNF)-α, IL-1β, IL-6, and IL-12. M1 macrophages have a high capacity for phagocytosis and the production of reactive oxygen species (ROS), and are involved in promoting inflammation and defense against microbial infections. M2 macrophages are activated in response to anti-inflammatory stimuli, including IL-4 and IL-13. They secrete anti-inflammatory cytokines, such as IL-10 and transforming growth factor (TGF)-β, along with other factors like arginase-1 and IL-1 receptor antagonist. M2 macrophages help to resolve inflammation, promote tissue healing, and clear cellular debris. The differing functions of these two macrophage subtypes produce distinct effects on surrounding cells and establish pro- or anti-inflammatory environments [24,25]. Thus, macrophages promote the formation of complex and unstable plaques to maintain a pro-inflammatory microenvironment [26,27,28]. At the same time, anti-inflammatory macrophages contribute to tissue repair and remodeling, as well as plaque stabilization [26,27,28].

p190RhoGEF is a GEF that plays a role in regulating Rho GTPases, which are critical in various cellular processes, including cell migration, proliferation, and cytoskeleton organization. While p190RhoGEF has been studied in other contexts, such as in cancer and cell motility, its direct role in atherosclerosis has not been thoroughly investigated or well established. Some proteins and signaling pathways that play a crucial role in atherosclerosis may intersect with those regulated by Rho GTPases and their associated factors. In this study, we examined whether p190RhoGEF over-expression in macrophages affects the atherosclerotic disease process in a mouse model: p190RhoGEF-TG mice were cross-bred with atherosclerosis-prone ApoE^−/−^ mice to obtain p190RhoGEF-TG mice with an ApoE^−/−^ background (TG/ApoE^−/−^). Thus, ApoE deficiency-driven spontaneous atherosclerosis was examined in standard rodent-chow-fed TG/ApoE^−/−^ mice. The findings of the current study suggest that over-expression of p190RhoGEF in mice with an ApoE^−/−^ background influences the functions of macrophages in a chronic inflammatory state, which could affect spontaneous atherosclerosis.

## 2. Results

### 2.1. Over-Expression of p190RhoGEF in Cells Bearing the Class II MHC Affects Phenotypes of ApoE^−/−^ Mice That Spontaneously Develop Atherosclerosis

To study the role of p190RhoGEF in vivo, we developed p190RhoGEF-TG mice, in which a transgene is specifically expressed in antigen-presenting cells bearing the class II MHC [7]. We have previously confirmed that the p190RhoGEF transgene in p190RhoGEF-TG mice was strongly expressed in B cells, DCs, and macrophages expressing class II MHC molecules [10,11,12]. To examine the role of these cells over-expressing p190RhoGEF in the development of atherosclerosis, p190RhoGEF-TG mice were cross-bred with ApoE^−/−^ mice that spontaneously developed atherosclerosis [18,19,21].

**Figure 1:**
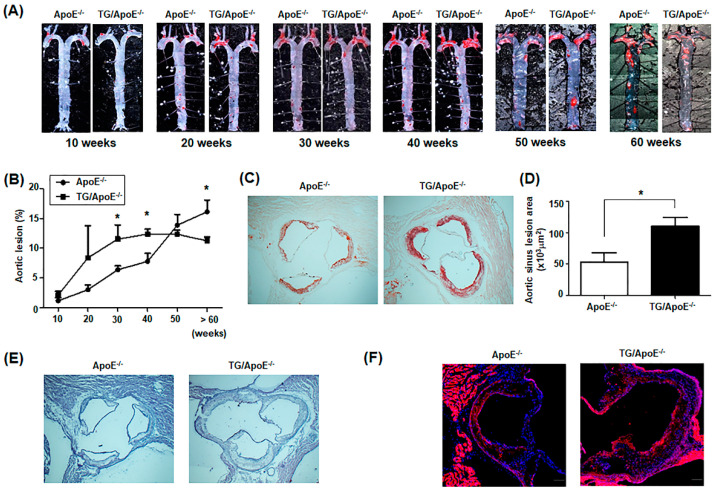
Atherosclerotic plaque formation in the aortae of ApoE^−/−^ and TG/ApoE^−/−^ mice fed a standard rodent chow diet. (**A**) Representative photomicrographs of atherosclerotic plaques in the aortae of ApoE^−/−^ and TG/ApoE^−/−^ mice at different age groups. Aortae isolated from mice at 10 to >60 weeks of age were fixed and stained with Oil Red O. (**B**) Age-dependent changes in aortic lesions in ApoE^−/−^ and TG/ApoE^−/−^ mice. Atherosclerotic aortic lesions were quantified by the percentage relative to the total aortic area. Data represent the mean of results from at least five mice in each age group (*n* = 6 at 10 and 20 weeks; *n* = 11 at 30 weeks; *n* = 5 at 40 and 50 weeks; *n* = 7 at >60 weeks). Significance was determined by unpaired *t* tests (* *p* < 0.05). (**C**) Representative Oil Red O staining of aortic sinuses from mice at 40 weeks of age. (**D**) Quantification of area of aortic sinus plaques from mice at 30 to 50 weeks of age. Data shown are the mean ± SEM (*n* = 5). Significance was determined by unpaired *t* tests (* *p* < 0.05). (**E**) Representative H&E-stained cross sections of aortic sinuses from mice at 50 weeks of age. (**F**) Representative α-SMA staining of aortic sinuses from mice at 50 weeks of age. Scale bars = 100 μm.

We examined atherosclerotic plaque formation in the aorta of ApoE^−/−^ and p190RhoGEF-TG mice with ApoE^−/−^ background (TG/ApoE^−/−^ mice) fed a standard rodent chow diet. Using Oil Red O staining, spontaneous atherosclerosis was compared in TG/ApoE^−/−^ and control ApoE^−/−^ mice at 10 to 60 weeks of age (Figure 1A). After 10 weeks of age, the TG/ApoE^−/−^ mice began to develop more plaques than the ApoE^−/−^ control mice did, and this increase became significant at 30–40 weeks of age (Figure 1B). Atherosclerotic plaques grew rapidly in the aortae of 10- to 30-week-old TG/ApoE^−/−^ mice and remained stable until 50 weeks of age, but then started to decrease in areal extent at 60 weeks (Figure 1B). In contrast, the aortic plaques in ApoE^−/−^ mice grew more slowly in younger mice, but continued to grow in the aged mice (Figure 1B). The areal extent of plaque formation in 50-week-old control ApoE^−/−^ mice approached that of TG/ApoE^−/−^ mice and exceeded that of TG/ApoE^−/−^ mice older than 60 weeks (Figure 1B). Increased atherosclerotic lesions were also seen in the aortic roots of 30- to 50-week-old TG/ApoE^−/−^ mice compared to the age-matched ApoE^−/−^ control mice (Figure 1C,D). Moreover, hematoxylin and eosin (H&E) staining and anti-alpha-smooth muscle actin (α-SMA) staining showed more intimal thickening and fibrous caps in the aortic roots of TG/ApoE^−/−^ mice compared to the age-matched ApoE^−/−^ mice (Figure 1E,F). These results suggest that class II MHC-driven over-expression of p190RhoGEF in TG/ApoE^−/−^ mice drives more severe and earlier-onset atherosclerotic plaque development followed by plaque regression compared to the control ApoE^−/−^ mice, which continue to show increasing plaque burden.

### 2.2. Immune Cell Profiles Are Similar in ApoE^−/−^ and TG/ApoE^−/−^ Mice

**Figure 2:**
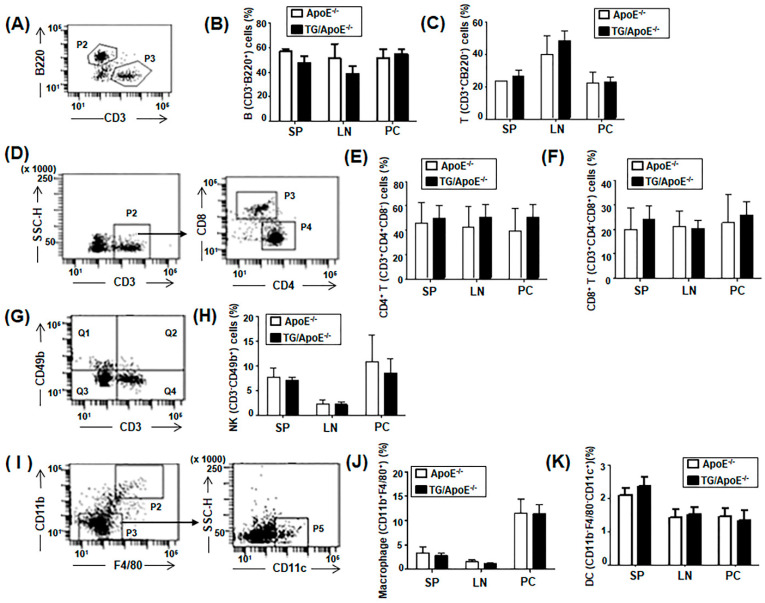
Immune cell profiles in ApoE^−/−^ and TG/ApoE^−/−^ mice. Total cells were isolated from the spleens (SP), lymph nodes (LN), and peritoneal cavities (PC) of ApoE^−/−^ and TG/ApoE^−/−^ mice aged 30 to 50 weeks. Cells were surface-labeled using different color combinations of fluorescence-conjugated antibodies against marker proteins of various immune cells, and were subjected to flow cytometric analysis. (**A**,**D**,**G**,**I**) Representative dot plots of fluorescence intensity and gating strategies are shown for B (P2, B220^+^CD3^−^) and T (P3, CD3^+^B220^−^) cells (**A**), CD3^+^CD8^+^ (P3) and CD3^+^CD4^+^ (P4) T cells (**D**), NK (Q1, CD3^−^CD49b^+^) cells (**G**), macrophages (P2, F4/80^+^CD11b^+^), and DC (F4/80^−^CD11b^−^CD11c^+^) (**I**). (**B**,**C**,**E**,**F**,**H**,**J**,**K**) The percentages of cells in the gated area are shown as the means ± SEM of five separate experiments. Significant comparisons were determined by unpaired *t* tests.

We previously published a study demonstrating that the over-expression of p190RhoGEF affects B cell activation and differentiation [10], which in turn enhances T cell immunity. These components of the adaptive immune system are known to be involved in the development of atherosclerosis as well [15,16,17]. We then characterized major immune cell lineage in the spleens, lymph nodes, and peritoneal cavities of ApoE^−/−^ control and TG/ApoE^−/−^ mice at 30 to 50 weeks of age. It was noted that the specific immune cell profiles observed in ApoE^−/−^ mice could vary depending on factors such as age, diet, and the stage of atherosclerosis development. Additionally, individual research studies may report varying results depending on their experimental designs and methodologies. We did not see any significant differences in the population of B and T cells, natural killer (NK) cells, DCs, and macrophages between age-matched ApoE^−/−^ control and TG/ApoE^−/−^ mice (Figure 2). These results suggest that class II MHC-driven over-expression of p190RhoGEF does not influence immune cell profiles in TG/ApoE^−/−^ mice.

### 2.3. Serum Concentrations of Pro-Inflammatory Cytokines Increased in TG/ApoE^−/−^ Mice

**Figure 3:**
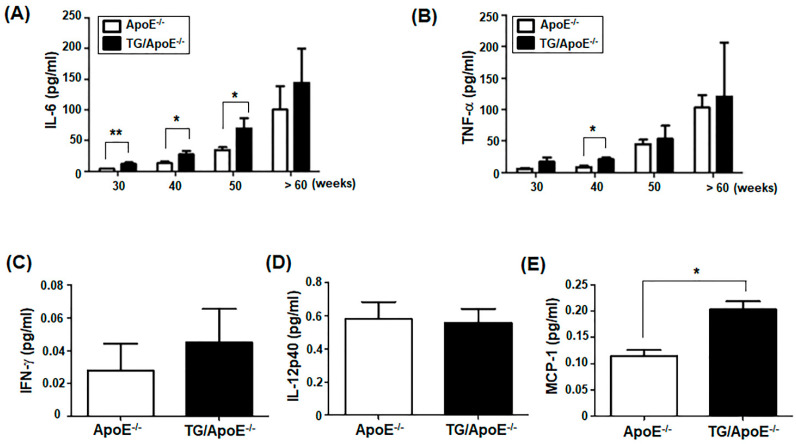
Serum pro-inflammatory cytokines in ApoE^−/−^ and TG/ApoE^−/−^ mice. Sera were obtained from mice aged 30 to >60 weeks. (**A**,**B**) Pro-inflammatory cytokines IL-6 and TNF-α were analyzed by ELISA. The data shown represent the mean ± SEM (*n* = 5 at 30 and 40 weeks; *n* = 4 at 50 and >60 weeks). (**C**–**E**) IFN-γ, IL-12p40, and the chemokine MCP-1 were analyzed using ELISA. The data shown represent the mean ± SEM (*n* = 3). Significance was determined by unpaired *t* tests (* *p* < 0.05, ** *p* < 0.01).

The number of circulating pro-inflammatory monocytes is significantly increased in animal models of atherosclerosis such as the ApoE^−/−^ mouse, and pro-inflammatory cytokines are secreted during atherosclerotic lesion progression [17,29,30]. We examined whether TG/ApoE^−/−^ mice produced increased pro-inflammatory cytokines. Serum concentrations of the pro-inflammatory cytokines IL-6 and TNF-α were compared in age-matched ApoE^−/−^ and TG/ApoE^−/−^ mice using an enzyme-linked immunosorbent assay (ELISA) (Figure 3A,B). At 30 to 50 weeks of age, TG/ApoE^−/−^ mice had increased serum concentrations of IL-6 and TNF-α compared with those in ApoE^−/−^ control mice. These changes in inflammatory cytokines might affect atherosclerotic plaque growth in TG/ApoE^−/−^ mice. At ages older than 60 weeks, the serum concentrations of IL-6 and TNF-α in ApoE^−/−^ control mice approached those in TG/ApoE^−/−^ mice. These changes in cytokines might affect rapid plaque growth in ApoE^−/−^ control mice older than 60 weeks. However, the plaque regression shown in TG/ApoE^−/−^ mice older than 60 weeks was not correlated with serum inflammatory cytokines. These results suggest that the interplay of various factors other than inflammatory cytokines can influence atherosclerotic plaque formation in mice older than 60 weeks. Further investigation was performed in TG and control ApoE^−/−^ mice at 30 to 50 weeks of age in consideration of the correlation of inflammatory cytokine production and plaque growth. We also examined IFN-γ and IL-12 production because these cytokines induce macrophage M1 subset polarization and pro-inflammatory Th1 cell differentiation, respectively. The concentrations of IFN-γ and IL-12 in serum were similar between the ApoE^−/−^ control and TG/ApoE^−/−^ mice (Figure 3C,D). Additionally, the production of a chemokine, i.e., monocyte chemoattractant protein (MCP)-1, which plays a crucial role in attracting monocytes/macrophages to the site of inflammation in atherosclerosis, was determined. The serum concentration of MCP-1 was significantly increased in TG/ApoE^−/−^ mice compared with that in ApoE^−/−^ control mice (Figure 3E). As shown in Figure 3, the serum concentrations of IFN-γ , IL-12, and MCP-1 were relatively small compared to those of IL-6 and TNF-α. Increased serum concentrations of IL-6, TNF-α, and MCP-1, but not IFN-γ and IL-12, in TG/ApoE^−/−^ mice suggest that proinflammatory cytokines IL-6 and TNF-α, produced by M1 subset macrophages, play significant roles in the inflammatory responses accompanying atherosclerotic plaque formation.

### 2.4. Macrophage Infiltration Is Increased in Aortic Sinus Plaques of TG/ApoE^−/−^ Mice

**Figure 4:**
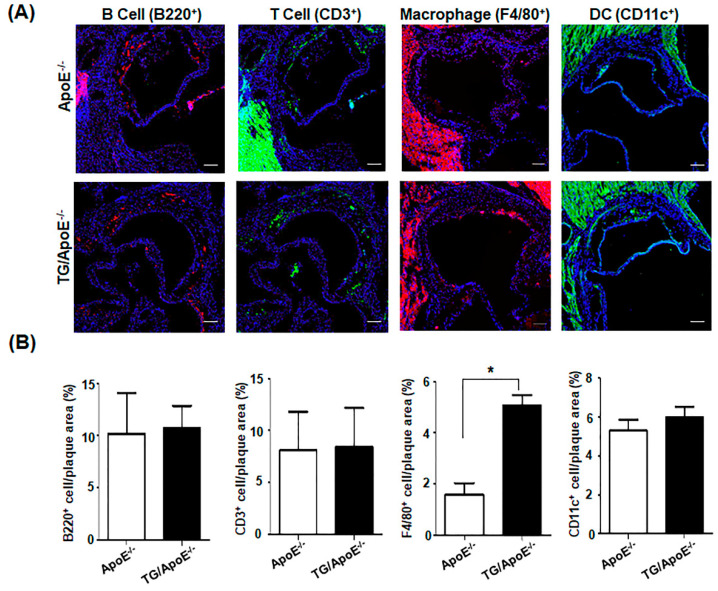
Immune cell infiltration into the aortic sinus plaques of ApoE^−/−^ and TG/ApoE^−/−^ mice. The hearts and proximal aortic roots were isolated from ApoE^−/−^ and TG/ApoE^−/−^ mice at 40 to 50 weeks of age and then frozen. The frozen sections were stained with fluorescence-conjugated Abs against marker proteins of various immune cells and DAPI (blue). (**A**) Representative images of immunohistochemistry results from the aortic roots of ApoE^−/−^ and TG/ApoE^−/−^ mice at 48 weeks of age are shown. B220 (red), CD3 (green), F4/80 (red), and CD11c (green) positive cells show B and T cells, macrophages, and DC, respectively. Scale bars = 100 μm. (**B**) Immune cell infiltration was quantified as the percentage of the area of stained cells within the plaque area. The data shown represent the means ± SEM from the tissue sections of three ApoE^−/−^ and TG/ApoE^−/−^ mice. Significance was determined using an unpaired *t* test (* *p* < 0.05).

It is widely accepted that circulating pro-inflammatory monocyte-derived cells are recruited to atherosclerotic lesion sites during vascular inflammation and subsequently differentiate into macrophages [17,22,23,30]. We examined whether the changes in development of spontaneous atherosclerosis observed in TG/ApoE^−/−^ mice could be explained by the degree of macrophage infiltration into the arterial wall. We stained cryo-sections of the aortic root with an antibody (Ab) that detects F4/80, a molecular marker of macrophages. Representative immunohistochemistry images of the arterial wall of ApoE^−/−^ and TG/ApoE^−/−^ mice at ~40 weeks of age are shown in Figure 4A. The area of F4/80^+^ cells within the lesion sites was significantly larger in the TG/ApoE^−/−^ mice than that in the ApoE^−/−^ mice (Figure 4B). We also analyzed B cells, T cells, and DCs within the lesion and did not find any significant difference between ApoE^−/−^ control and TG/ApoE^−/−^ mice (Figure 4A,B). These results suggest that infiltrating macrophages play a role in the differences in progression of spontaneous atherosclerotic lesions in ApoE^−/−^ and TG/ApoE^−/−^ mice.

### 2.5. Macrophages of TG/ApoE^−/−^ Mice Produce Higher Levels of Pro-Inflammatory Cytokines than Do Control ApoE^−/−^ Mice

**Figure 5:**
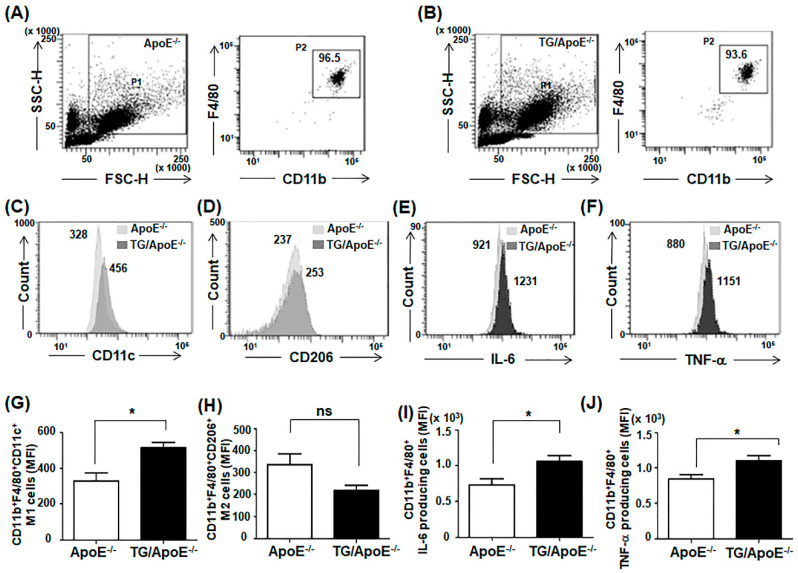
M1 subset population and pro-inflammatory cytokine production in peritoneal macrophages of ApoE^−/−^ and TG/ApoE^−/−^ mice. Peritoneal macrophages were harvested from 40- to 50-week-old mice. To assess the M1 subset population, peritoneal macrophages were labeled with FITC-conjugated anti-CD11c mAb along with PE-conjugated anti-CD11b and PerCP-Cy5.5-conjugated anti-F4/80 or APC-conjugated CD206 mAbs. To analyze macrophages producing IL-6 or TNF-α, peritoneal macrophages were treated with GolgiStop^TM^, and intracellular cytokines were stained with APC-conjugated anti-IL6 or anti-TNF-α along with PE-conjugated anti-CD11b and PerCP-Cy5.5-conjugated anti-F4/80 mAbs. Three-color flow cytometric analyses were then conducted. (**A**,**B**) Representative dot plots show SSC vs. FSC (live cells, gated in P1) and F4/80 vs. CD11b staining (macrophages from live cell population, gated in P2). The numbers indicate the percentage of macrophages among live cells. (**C**–**F**) The levels of expression of the M1 and M2 markers (CD11c and CD206) and the production of intracellular cytokines IL-6 and TNF-α in the macrophages (P2) of ApoE^−/−^ and TG/ApoE^−/−^ mice are compared as histograms. The numbers indicate the mean fluorescence intensities (MFI). (**G**–**J**) The MFI are compared, and the data from three independent experiments are presented as the mean ± SEM. Statistical significance was analyzed using unpaired *t* tests (* *p* < 0.05).

Given the increase in pro-inflammatory cytokines in the serum of TG/ApoE^−/−^ mice compared with that of ApoE^−/−^ control mice (Figure 3A,B), we investigated the peritoneal macrophages of the two groups of mice. Flow cytometry analyses of macrophages were performed by staining harvested peritoneal macrophages for F4/80 and CD11b (Figure 5A,B). The total number of cells expressing F4/80 and CD11b was similar in ApoE^−/−^ and TG/ApoE^−/−^ mice (Figure 5A,B), but the proportion of the M1 subset (CD11b^+^F4/80^+^CD11c^+^) was significantly greater among peritoneal macrophages of TG/ApoE^−/−^ mice than ApoE^−/−^ mice (Figure 5C,G). The M2 subset population (CD11b^+^F4/80^+^CD206^+^) was similar or reduced in TG/ApoE^−/−^ mice compared to in ApoE^−/−^ mice, although the difference was not statistically significant (Figure 5D,H).

The M1 subset of peritoneal macrophages produces high levels of inflammatory cytokines such as TNF-α and IL-6 [27,28]. We next examined whether peritoneal macrophages from TG/ApoE^−/−^ mice that contain a greater proportion of M1-subtype cells produce more inflammatory cytokines than macrophages from ApoE^−/−^ mice do. Flow cytometric assessment of intracellular staining for IL-6 and TNF-α indicated greater production of these cytokines in macrophages from TG/ApoE^−/−^ mice compared with those from ApoE^−/−^ mice (Figure 4E,I,F,J).

### 2.6. Macrophages of TG/ApoE^−/−^ Mice Show Enhanced Responses to LPS

**Figure 6:**
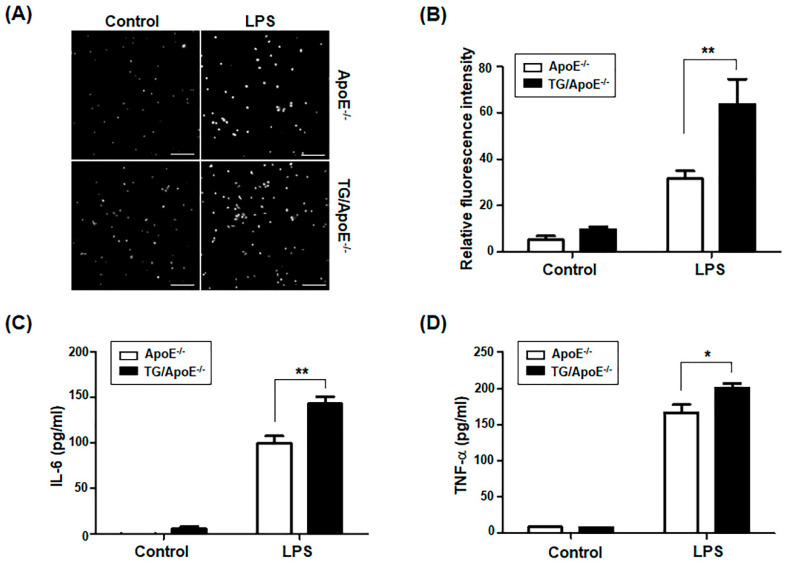
LPS responses in the peritoneal macrophages of ApoE^−/−^ and TG/ApoE^−/−^ mice. Peritoneal macrophages were isolated from 30- to 40-week-old mice. (**A**) Images show LPS-mediated ROS production in the macrophages of ApoE^−/−^ and TG/ApoE^−/−^ mice. Macrophages (1 × 10^6^) that were loaded with 5 μM 2′,7′-dichlorofluoscein diacetate (DCFDA) for 15 min were stimulated with LPS (1 μg/mL). Representative microscopic fields of DCF fluorescence from the cells after 30 min of stimulation with either PBS (control) or LPS. Scale bars = 100 μm. (**B**) The DCF fluorescence intensity of macrophages from ApoE^−/−^ and TG/ApoE^−/−^ mice after 30 min of stimulation with LPS. The data are the averaged intensities from three repeated experiments (intensities of cells in three microscopic fields were averaged in each experiment). The data are presented as the mean ± SEM. Statistical significance was analyzed by two-way ANOVA with Bonferroni post-tests. Significant comparisons are noted as ** *p* < 0.01. (**C**,**D**) Peritoneal macrophages from ApoE^−/−^ and TG/ApoE^−/−^ mice were cultured either in media alone (control) or with LPS (100 ng/mL) overnight. The levels of IL-6 and TNF-α in the supernatants were compared by ELISA. The data are the mean ± SEM of three independent experiments with triplet sample settings. Statistical significance was analyzed by two-way ANOVA with Bonferroni post-tests. Significant comparisons are noted as * *p* < 0.05, ** *p* < 0.01.

Studies in ApoE^−/−^ and LDLR^−/−^ mice have shown that chronic inflammation triggered by metabolic mediators such as cholesterol and ceramide, as well as due to persistent bacterial infection, is associated with an increased incidence of atherosclerosis [16,18,19,20,31,32]. We assessed the response of peritoneal macrophages from TG/ApoE^−/−^ and ApoE^−/−^ control mice stimulated with bacterial lipopolysaccharides (LPS). To test the ability to produce reactive oxygen species (ROS), macrophages were labeled using 2′,7′-dichloro-dihydrofluorescein (DCF) dye that fluoresces in response to intracellular ROS. Representative fluorescence images of cells after 30 min of stimulation with LPS are shown in Figure 6A; the fluorescence intensities are compared quantitatively in Figure 6B. After LPS activation, ROS production was significantly increased in peritoneal macrophages harvested from TG/ApoE^−/−^ mice compared with those from ApoE^−/−^ mice. Secretion levels of the pro-inflammatory cytokines IL-6 and TNF-α were measured using an ELISA. Cytokine secretion in response to LPS was significantly greater in TG/ApoE^−/−^ macrophages than in control ApoE^−/−^ macrophages (Figure 6C,D). These results were consistent with the increase in the M1 subset and in the production of pro-inflammatory cytokines in peritoneal macrophages of TG/ApoE^−/−^ mice, as shown in Figure 5.

## 3. Discussion

We previously identified dramatically increased expression of p190RhoGEF in B cells following CD40 stimulation [8]. We showed that increased expression of p190RhoGEF during CD40-mediated B cell activation was correlated with the expression of specific surface molecules and transcriptional regulators required for B cell maturation and differentiation [9]. We also examined the role of p190RhoGEF in vivo using TG mice, in which expression of p190RhoGEF was driven by the class II MHC invariant chain (Ii) promoter. Transgenes were specifically expressed in antigen-presenting cells with class II MHC, including B cells, macrophages, and DCs. In contrast to the role of p190RhoGEF over-expression in B cells [10], we demonstrated that over-expression of p190RhoGEF in DCs and macrophages negatively regulates responses to LPS [11,12].

Our studies of macrophages from TG mice showed that over-expression of p190RhoGEF resulted in distinct phenotypic and functional changes, such that macrophages were roundish in shape and showed reduced responses to inflammation [12]. These changes in p190RhoGEF over-expressed TG macrophages were removed by the inhibition of RhoA activation [12]. RhoA activation followed by regulation of the actin cytoskeleton has been shown to change macrophage shapes and functions [33,34,35]. The reduction in M1 polarization during peritoneal inflammation and in responses to LPS stimulation in TG macrophages led us to further investigate the role of TG macrophages in a model of inflammatory disease.

Atherosclerosis is considered a chronic inflammatory disease in which macrophages play a decisive role at all stages [26,27,28]. It has been noted that ApoE deficiency in mice being fed a standard rodent chow diet results in spontaneous atherosclerosis at three to four months of age, with lesions appearing mostly in the proximal aorta, where they are characterized by vascular inflammation associated with infiltration of macrophages and other immune cells [16,17]. To examine whether p190RhoGEF over-expression in macrophages affects the progression of atherosclerotic lesions, p190RhoGEF-TG mice were cross-bred with atherosclerosis-prone ApoE^−/−^ mice (TG/ApoE^−/−^).

Atherosclerotic plaques grew rapidly in the aorta of TG/ApoE^−/−^ mice until 50 weeks of age and started to decrease at ages older than 60 weeks (Figure 1). In contrast, aortic plaques in ApoE^−/−^ mice grew more slowly in younger mice, but continued to grow as the mice aged; more plaques were found at ages older than 60 weeks compared to TG/ApoE^−/−^ mice (Figure 1). These results can be explained by the interplay of various factors that influence atherosclerosis progression and regression. The over-expression of p190RhoGEF in macrophages in TG/ApoE^−/−^ mice leads to increased RhoA activation and enhanced macrophage infiltration into atherosclerotic lesions (as shown in Figure 4), which accelerates plaque formation. As a result, TG/ApoE^−/−^ mice showed a more severe and earlier onset of atherosclerosis compared to the control ApoE^−/−^ mice. The exact mechanism of the regression shown in TG/^−/−^mice after 50 weeks might involve several factors, such as changes in macrophage polarization by the continued expression of RhoA-specific GEF and, potentially, the regulation of RhoA signaling in macrophages by regulatory mechanisms to downregulate RhoA activation in macrophages. In contrast, ApoE^−/−^ mice continue to show increasing plaque burden due to ongoing imbalances in lipid metabolism and a lack of the protective effects of ApoE on macrophage phenotypes.

Analyses of the immune systems of age-matched ApoE^−/−^ and TG/ApoE^−/−^ mice revealed that the populations of B and T cells, and their subsets; NK cells; DCs; and macrophages were similar in the lymph nodes, spleens, and peritoneal fluids (Figure 2). These results suggest that class II MHC-driven over-expression of p190RhoGEF does not influence immune cell profiles in TG/ApoE^−/−^ mice, and that the differential atherosclerotic plaque formation observed in ApoE^−/−^ and TG/ApoE^−/−^ mice is not due to differences in the immune cell profiles.

To better understand the inflammatory mechanisms associated with atherosclerotic plaque formation in ApoE^−/−^ and TG/ApoE^−/−^ mice, the levels of several inflammatory cytokines and chemokines in the sera of these mice were analyzed. Increased serum concentrations of the pro-inflammatory cytokines IL-6 and TNF-α were clearly shown in TG/ApoE^−/−^ mice at 30 to 50 weeks of age compared to age-matched ApoE^−/−^ mice (Figure 3). Serum concentrations of these cytokines in ApoE^−/−^ control mice approached those in TG/ApoE^−/−^ mice at ages older than 60 weeks. Increases in these cytokines might affect rapid plaque growth in ApoE^−/−^ control mice older than 60 weeks. However, the plaque regression shown in TG/ApoE^−/−^ mice older than 60 weeks was not correlated with serum inflammatory cytokines. These results suggest that the interplay of various factors other than inflammatory cytokines can influence atherosclerotic plaque formation in mice older than 60 weeks. Further studies including analyses of the changes in lipid profiles, macrophage polarization, as well as RhoA signaling in macrophages could possibly explain the difference in the plaque progression and regression between ApoE^−/−^ and TG/ApoE^−/−^ mice. Current studies have focused on the effect of p190RhoGEF over-expression in the progression of atherosclerotic plaque development and inflammatory cytokine production in TG/ApoE^−/−^ mice.

Some major immune cells were quantified in the aortic sinus plaques of age-matched ApoE^−/−^ and TG/ApoE^−/−^ mice. B cells, T cells, and DCs were similarly identified, but a higher infiltration of macrophages was shown in TG/ApoE^−/−^ mice than in ApoE^−/−^ control mice at 30 to 50 weeks of age (Figure 4). A significant increase in MCP-1 was also shown in the sera of TG/ApoE^−/−^ mice at 30 to 50 weeks of age compared to age-matched ApoE^−/−^ control mice (Figure 3). This pattern of macrophage infiltration coincides with increased atherosclerotic plaque formation in the aortae of TG/ApoE^−/−^ mice (Figure 1). Moreover, increased inflammatory cytokines in the macrophages of TG/ApoE^−/−^ mice at 30 to 50 weeks of age strongly suggest that increased concentrations of serum inflammatory cytokines in the TG/ApoE^−/−^ mice were due to increased cytokine production by circulating macrophages and, possibly, those of the M1 subtype (Figure 5). In accordance with the in vivo findings, macrophages isolated from TG/ApoE^−/−^ mice showed enhanced responses to LPS, such as ROS production and secretion of the inflammatory cytokines IL-6 and TNF-α (Figure 6).

We found that over-expression of the p190RhoGEF transgene leads to opposite results with regard to macrophage functions when present with or without co-expression of the ApoE gene. Compared with the macrophages of TG/ApoE^−/−^ mice, pro-inflammatory M1-subset polarization was reduced in TG macrophages during peritoneal inflammation [12]. The macrophages of TG and TG/ApoE^−/−^ mice also showed opposite responses with respect to the production of ROS and inflammatory cytokines following LPS stimulation (Figure 6). These opposite macrophage responses in TG and TG/ApoE^−/−^ mice can be explained by the regulation of RhoA activation [35,36,37,38,39]. These opposite results in macrophage polarization observed in TG/ApoE^−/−^ compared to TG mice can be attributed to the interplay of multiple factors, including the presence or absence of ApoE deficiency and the over-expression of RhoA-specific GEF in macrophages. The presence or absence of ApoE deficiency can profoundly impact the inflammatory context in which RhoA-specific p190RhoGEF is over-expressed in macrophages. This inflammatory context can modulate the responses of macrophages to RhoA signaling, leading to different polarization outcomes. Thus, the observed opposite results in macrophage polarization between TG/ApoE^−/−^ and TG mice can be explained by the interaction between RhoA-specific p190RhoGEF over-expression, ApoE deficiency, and the inflammatory environment. The absence of ApoE expression likely creates a pro-inflammatory milieu that interacts with RhoA signaling to promote M1 macrophage polarization in TG/ApoE^−/−^ mice, while in the presence of ApoE expression, TG mice show a reduction in M1 macrophages due to different regulatory mechanisms.

Macrophages play a key role in many chronic inflammatory diseases. Because macrophages are highly modifiable and heterogeneous, they play essential roles in pro- or anti-inflammatory environments, consequently influencing the surrounding cells and regulating immune responses [23,24,25]. Macrophages also play various important roles in atherosclerosis, promoting the formation of complex and unstable plaques by maintaining the pro-inflammatory microenvironment, while anti-inflammatory macrophages contribute to tissue repair and remodeling, as well as plaque stabilization [26,27,28]. Therefore, macrophages are attractive targets for the development of anti-atherosclerotic therapy. In this study, we showed that over-expression of p190RhoGEF in mice with an ApoE^−/−^ background influences the functions of macrophages in a chronic inflammatory state. As mentioned earlier, the over-expression of p190RhoGEF in macrophages in TG/ApoE^−/−^ mice leads to increased RhoA activation, consequent pro-inflammatory M1 phenotype polarization, and enhanced macrophage infiltration into atherosclerotic lesions, which accelerates plaque formation. Although these results may not provide a direct link between the role of p190RhoGEF and atherosclerosis, they imply potential involvement of p190RhoGEF in the atherosclerotic process. The macrophage modulation by p190RhoGEF during the atherosclerotic process, including plaque regression, as shown in TG/ApoE^−/−^ mice, remains to be investigated further.

The results in this study help us to better understand the complex and dynamic nature of atherosclerosis. They highlight the crucial roles of macrophages and RhoA signaling in atherosclerosis development and regression. The observed plaque regression in TG/ApoE mice demonstrates the potential for therapeutic interventions targeting RhoA or macrophage polarization to halt or reverse atherosclerosis. While this study was conducted in mice, the findings may have potential clinical implications. Targeting RhoA-specific p190RhoGEF expression or RhoA signaling in macrophages could be explored as a therapeutic strategy to modulate atherosclerosis progression in humans. Additionally, promoting macrophage polarization towards an anti-inflammatory phenotype could be investigated as a means to facilitate plaque regression and promote vascular health in individuals with atherosclerosis.

## 4. Materials and Methods

### 4.1. Mice

The generation of p190RhoGEF-TG mice occurred as described previously [11]. To generate TG/ApoE^−/−^ mice, p190RhoGEF-TG mice were cross-bred with ApoE^−/−^ mice (provided by Dr. Y. S. Bae, Ewha Womans University, Korea). Both strains of mice were established on the C57BL/6 genetic background. The p190RhoGEF transgene was confirmed by PCR with primer I (5′-GTG CCT GCT TCT AGA ACC GTC-3′) and primer II (5′-GCA ACC TTC CTG CAA CAG AGC-3′). The ApoE gene was determined by PCR with primer I (5′-AGA ACT GAC GTG AGT GTC CA-3′), primer II (5′-GTT CCC AGA AGT TGA GAA GC-3′), and primer III (5′-GCT TCC TCG TGC TTT ACG GTA-3′) [40]. Mice were fed a normal chow diet (Teklad™ 2018C, Envigo, Indianapolis, IN, USA) and administered filtered water in an autoclaved water bottle. Mice were bred and maintained under specific pathogen-free conditions (maintained disease-free by health monitoring testing from QM diagnostics using the Vivium sampling kit) at the animal facility of Ewha Laboratory Animal Genomic Center in accordance with the institutional guidelines. All animal experiments were performed in compliance with Guide for the Care and Use of Laboratory Animals from the Korean government and the U.S. Department of Health and Human Services. All mouse protocols were approved by the Ewha Institutional Animal Care and Use Committee.

### 4.2. Antibodies and Reagents

The following mAbs were purchased from eBioscience, Inc. (Santa Clara, CA, USA): PerCP-Cyanine5.5- or biotin-conjugated rat anti-mouse F4/80 (BM8), PE-conjugated rat anti-mouse CD11b (M1/70), FITC-conjugated rat anti-mouse CD11c (N418), and allophycocyanin (APC)-conjugated rat anti-mouse TNF-α (MP6-XT22). The APC-conjugated rat anti-mouse IL-6 (MP5-20F3) and CD206 (MMR) mAbs were obtained from BioLegend (San Diego, CA, USA). Rabbit Alexa Fluor^®^ 647 mAb to alpha smooth muscle actin (α-SMA) was obtained from abcam (Cambridge, UK). Isotype-control IgGs were purchased from Jackson ImmunoResearch Laboratories, Inc. (West Grove, PA, USA), and PE-Cy5-conjugated streptavidin, GolgiStop™ (monensin), and Cytofix/Cytoperm™ reagent were obtained from BD Biosciences (San Jose, CA, USA). Ready-SET-Go cytokine ELISA kits for mouse TNF-α, IL-6, IFN-γ, p40, and MCP-1 were obtained from eBioscience, Inc. The DAPI was purchased from Roche Diagnostics GmbH (Mannheim, Germany). The LPS (Escherichia coli, serotype 055:B5), bovine serum albumin (BSA), paraformaldehyde (PFA), Harris hematoxylin, eosin Y, and poly-L-lysine were purchased from Sigma-Aldrich Co. (St. Louis, MO, USA). The Tissue-Tek^®^ optimal cutting temperature (OCT) compound was purchased from Sakura Finetechnical Co., Ltd. (Tokyo, Japan), Oil Red O was obtained from Abcam Plc. (Cambridge, UK), and 2′, 7′-dichloro-dihydrofluorescein diacetate (DCFDA) was purchased from Molecular Probes (Eugene, OR, USA).

### 4.3. Isolation of Peritoneal Macrophages

Cells were harvested from the peritoneal cavity by injecting and recovering 1 mL of sterile PBS, then resuspended in RPMI culture medium. Macrophages were allowed to adhere to the culture dish by incubating overnight at 37 °C in a 5% CO_2_ incubator, and were then washed twice with PBS to remove non-adherent cells. Macrophages were collected using a scraper and washed twice with PBS prior to all experiments.

### 4.4. Analysis of Atherosclerotic Lesions

Mouse aorta isolation, Oil Red O staining, and quantification of the atherosclerotic burden were performed by reference to previously described protocols [41,42]. Mouse aortae were isolated from the proximal ascending aorta to abdominal aorta, and adventitial fat was removed. The aortae were opened longitudinally to expose the lumen. Isolated aortae were fixed with 4% PFA overnight at 4 °C. After being washed with PBS, the fixed aortae were stained with Oil Red O for 2 h and then decolorized with 60% isopropyl alcohol for 30 min at room temperature. Images were captured with a Leica DFC290 camera attached to a Leica S8AP0 dissecting microscope (Leica microsystems GmbH, Wetzlar, Germany). The total area and lesion area were calculated using Image J software (1.6.0). The results were expressed as percentage of the stained area relative to the area of the entire aorta.

### 4.5. Histochemistry and Immunofluorescence Staining for Aortic Cryosection

Histochemistry and immunofluorescence staining were performed as previously described [11,41,42]. The hearts and proximal aortic roots of ApoE^−/−^ and TG/ApoE^−/−^ mice were removed and embedded in OCT compound by quick freezing with liquid nitrogen. Frozen tissues were stored at −80 °C. A cryostat (Leica microsystems GmbH, Wetzlar, Germany) was used to obtain consecutive cryosections 8 μm thick from the aortic sinus; the sections were obtained by cutting the tissues on a cryostat, then mounted onto poly-L-lysine-coated slides. The sections were air-dried for 10 min before fixing in ice-cold acetone for 20 min; the sections were then air-dried again and stored at −20 °C. The sections were rehydrated with PBS and blocked with 3% BSA in PBS for 20 min at room temperature. For histology, the sections were subjected to hematoxylin and eosin (H&E) or Oil red O staining. The samples were analyzed using a Zeiss Axiovert 200 (4×) (Carl Zeiss, Inc., Oberkochen, Germany). For immunofluorescence staining, the sections were stained with biotin-conjugated rat anti-mouse F4/80 or B220 Ab overnight at 4 °C, followed by PE-Cy5-conjugated streptavidin for 30 min at room temperature and FITC-conjugated rat anti-mouse CD3 or CD11c Ab, Alexa Fluor^®^ 647 α-SMA Ab for 2 h. DAPI staining was performed shortly before embedding in 50% glycerol and covering with a coverslip. The samples were analyzed using a Nikon eclipse TS2R fluorescence microscope along with NIS-Elements BR 5.01.00 software (Tokyo, Japan) at Ewha Fluorescence Core Imaging Center. The percentages of B220^+^, CD3^+^, F4/80^+^, or CD11c^+^ cell-infiltrated areas among the sinus plaque areas were determined using Image J analysis software.

### 4.6. Flow Cytometry

Single-cell suspensions of isolated macrophages (2 × 10^5^/mL) were washed, blocked with the appropriate sera, and stained for specific cell-surface markers as described previously [9]. For the staining of intracellular cytokines, cells were fixed and permeabilized using a Cytofix/Cytoperm™ reagent, and intracellular IL-6 and TNF-α were stained using APC-conjugated Abs. Standard flow-cytometric analyses were performed for these cells. The data were acquired using an LSR Fortessa™ cell analyzer and BD FACSDiva software v8.0.1 (BD Biosciences, San Jose, CA, USA) at Ewha Fluorescence Core Imaging Center. Fluorescence signals were analyzed as dot plots of fluorescence intensity. The cell counts and mean fluorescence intensity (MFI) in the gated area are represented graphically in the figures.

### 4.7. Measurements of ROS

Intracellular ROS were measured using a fluorescent probe, 2′,7′-dichloro-dihydrofluorescein diacetate (DCFDA) [43]. Peritoneal macrophages (1 × 10^6^) were incubated in serum-free media for 1 h at 37 °C, then stimulated with LPS (1 μg/mL) for 30 min. Then, DCFDA (5 μM) was added, and the cells were incubated for 15 min before fluorescent cells were measured using a Nikon Eclipse Ts2R-FL microscope with a 20X objective lens.

### 4.8. ELISA

Ready-SET-Go cytokine ELISA sets were used according to the manufacturer’s instructions [12]. Macrophages (2.5 × 10^4^) were stimulated with LPS (100 ng/mL) in 200 μL of RPMI medium in a 96-well U-bottomed culture plate for 24 h. The culture supernatants and sera collected were added to each well of the 96-well flat-bottomed ELISA plates that were prepared for the assay. At the end of the assay, the plates were analyzed at 450 nm using a microplate reader, Spectra MAX 190 (Molecular Devices, San Jose, CA, USA).

### 4.9. Statistical Analysis

Comparisons between the samples were performed using an unpaired *t*-test. Comparisons of multiple conditions were performed using two-way ANOVA with post hoc tests. All statistical analyses were performed using Prism 5 software (GraphPad Software, Inc., San Diego, CA, USA). Values of *p* < 0.05 were considered significant. Asterisks mark significant differences between the different groups (* *p* < 0.05; ** *p* < 0.01).

## Data Availability

Not applicable.
